# Surgery to avoid fatal complications and secure radicality after definitive chemoradiotherapy for clinical T4N2M0 stage IIIB non-small cell lung cancer: a case report

**DOI:** 10.1186/s40792-019-0768-5

**Published:** 2020-01-13

**Authors:** Yuriko Yagi, Ken Kodama, Toru Momozane, Yukio Kimura, Masashi Takeda, Hiroki Kishima

**Affiliations:** 1 Department of Thoracic Surgery, Kinki Chuo Chest Medical Center, 1180 Nakazone-cho, Kita-ku, Sakai, Osaka, 591-8555 Japan; 2 Department of Thoracic Surgery, Yao Municipal Hospital, Osaka, Japan; 3 Department of Pathology, Yao Municipal Hospital, Osaka, Japan; 4 Department of Surgery, Kishima Hospital, Osaka, Japan

**Keywords:** Lung cancer, Chemoradiotherapy, Hemoptysis, Extended surgery, Left atrial resection

## Abstract

**Background:**

Chemoradiotherapy (CRT) is the standard treatment for c-stage IIIB non-small cell lung cancer (NSCLC); however, patients who respond to CRT are at risk of developing fatal complications such as massive hemoptysis or infection. In such cases, surgery is an alternative option. Currently, there are limited reports on surgery for complications arising during definitive CRT for locally advanced NSCLC. We report a case of hemoptysis after definitive CRT for c-T4N2M0 stage IIIB NSCLC that was successfully treated with lower bilobectomy combined with left atrial resection.

**Case presentation:**

A 72-year-old man with c-T4N2M0 stage IIIB NSCLC with left atrial invasion developed hemoptysis during CRT, which was discontinued to control hemoptysis. Chest computed tomography revealed a regressed and cavitated tumor. Three weeks after discontinuation of CRT, surgery was performed to avoid fatal complications and secure radicality. We performed lower bilobectomy combined with partial left atrial resection, which was performed using an automatic tri-stapler. The bronchial stump was covered with an omental flap. The resected specimen pathologically showed complete response with fistula between the intermediate bronchus and necrotic cavity in the tumor. His postoperative course was uneventful, and the patient was disease free at 10 months after surgery.

**Conclusions:**

We successfully performed surgery after definitive CRT in a patient with c-T4N2M0 stage IIIB NSCLC. Partial left atrial resection was safely performed with an automatic tri-stapler. A complete pathological response to CRT was achieved. In a case with a chance of complete (R0) resection, when the risk of developing fatal complications might outweigh the risk of post-CRT surgery perioperative complications, surgery should be considered as a treatment option.

## Background

The standard treatment for c-stage IIIB non-small cell lung cancer (NSCLC) is chemoradiotherapy (CRT). However, patients undergoing CRT are at risk of developing fatal complications, including massive hemoptysis or infection, due to tumor cavitation and fistula formation between the tumor and surrounding organs. The incidence of fatal pulmonary hemorrhage in patients with major cavitation on baseline chest computed tomography (CT) and squamous cell histology is reported to be 33.3% [1]. In such situations, post-CRT surgery is one of the treatment options [2]. Although the efficacy and safety of surgery after CRT has not been widely studied in lung cancer, a report concluded the median overall survival after salvage surgery to be 9–46 months and 5-year survival rate to be 20–75%, which are relatively good results [3]. In stage IIIB cases, the 5-year survival rate after post-CRT surgery was 42% for patients with no mediastinal lymph node involvement at the time of surgery and the rate of postoperative death was 7% [4]. Thus, selected patients might benefit from post-CRT surgery.

Herein, we report a case treated with right lower bilobectomy combined with left atrial resection for c-T4N2M0 NSCLC, which developed hemoptysis during definitive CRT. We highlight the surgical procedures employed to avoid intraoperative and postoperative complications.

## Case presentation

**Fig. 1:**
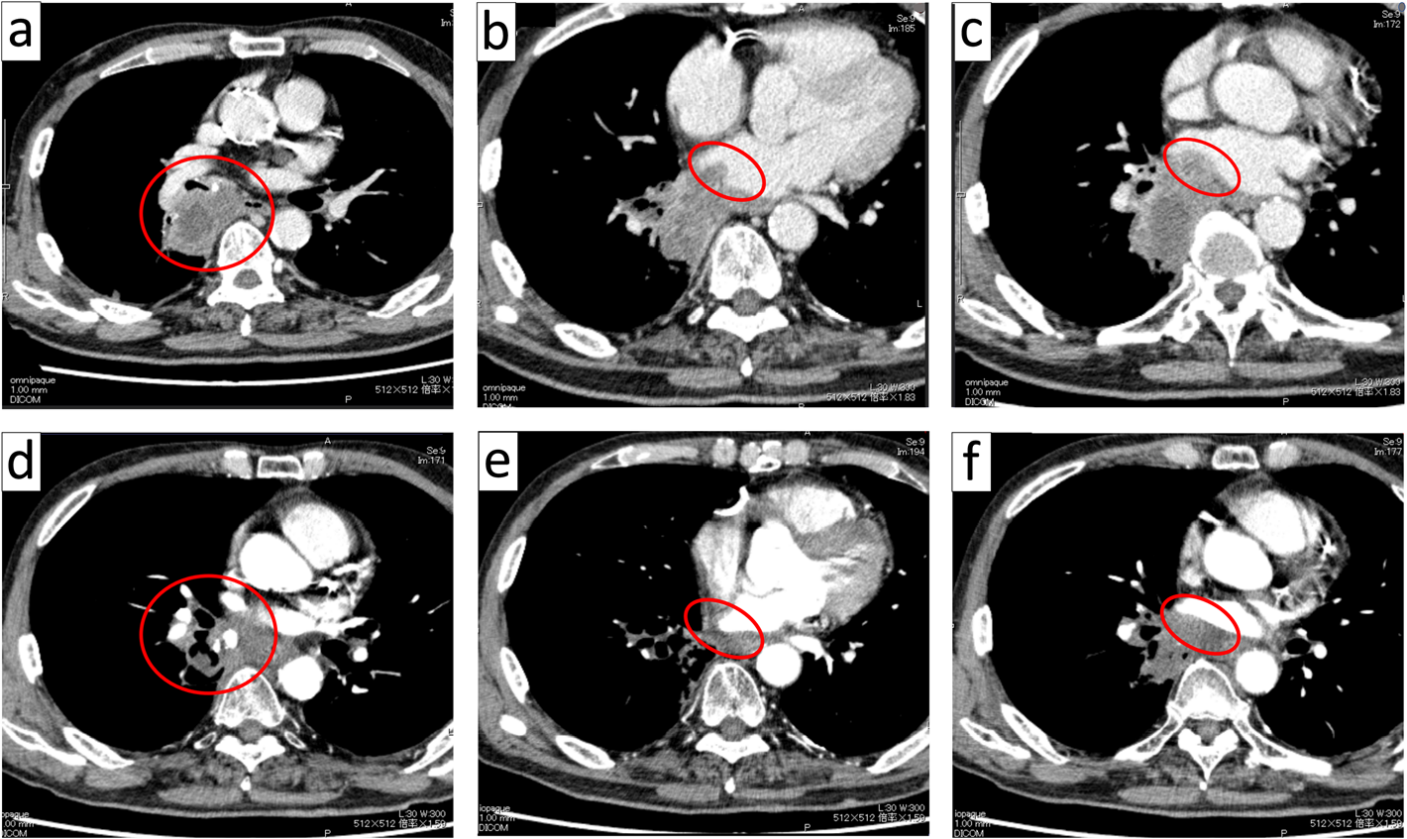
Chest computed tomography (CT) before chemoradiotherapy (CRT) (**a**–**c**) and before surgery (**d**–**f**)

A 72-year-old man, who was a former smoker for 76 pack-years, presented with dry cough persisting for 2 months. Chest CT revealed a 5.8-cm tumor in the right lower lobe (S 6/7 segment) fused with the enlarged hilar lymph nodes and mediastinal #7 lymph nodes (Fig. 1a). The mediastinal #4 lymph nodes were also enlarged. The tumor extended into the left atrium via the right inferior pulmonary vein (Fig. 1b). The left atrium was compressed by the tumor, which showed poor margins, suggesting direct invasion of the left atrial wall (Fig. 1c). Transbronchial lung biopsy failed to obtain sufficient material to make a diagnosis because of the bleeding tendency of the tumor. Based on the chest CT along with a serum carcinoembryonic antigen value of 12.3 ng/mL, we made a clinical diagnosis of right lower lobe c-T4N2M0 stage IIIB NSCLC. CRT was administered (weekly carboplatin [AUC2] plus paclitaxel [40 mg/m^2^] with concurrent thoracic radiotherapy; daily dose, 2 Gy).

After five courses of the regimen with total irradiation of 48 Gy, the patient developed hemoptysis. Therefore, we had to discontinue CRT. Chest CT revealed a cavitated tumor (Fig. 1d), which likely caused the hemoptysis. The right inferior pulmonary vein remained obstructed by the tumor (Fig. 1e). However, tumor extension into the left atrium had regressed (Fig. 1f). Preoperative echocardiogram revealed that the tumor protruding into the left atrium had regressed and that the left atrial wall motion had improved compared with the results of the echocardiogram performed before CRT.

Hemoptysis was controlled by treating with tranexamic acid and antibiotics, and discontinuation of CRT and aspirin prescribed for post-coronary stenting status. However, we considered that the newly developed cavity might develop massive hemoptysis in the future. Based on those findings, we decided to surgically control the life-threatening hemoptysis and achieve radical resection. The patient complicated chemotherapy-induced neutropenia, thrombocytopenia, and radiation-induced dermatitis on his back where the surgical wound would be placed. Therefore, we waited for 3 weeks to go for surgery after the discontinuation of CRT.

**Fig. 2:**
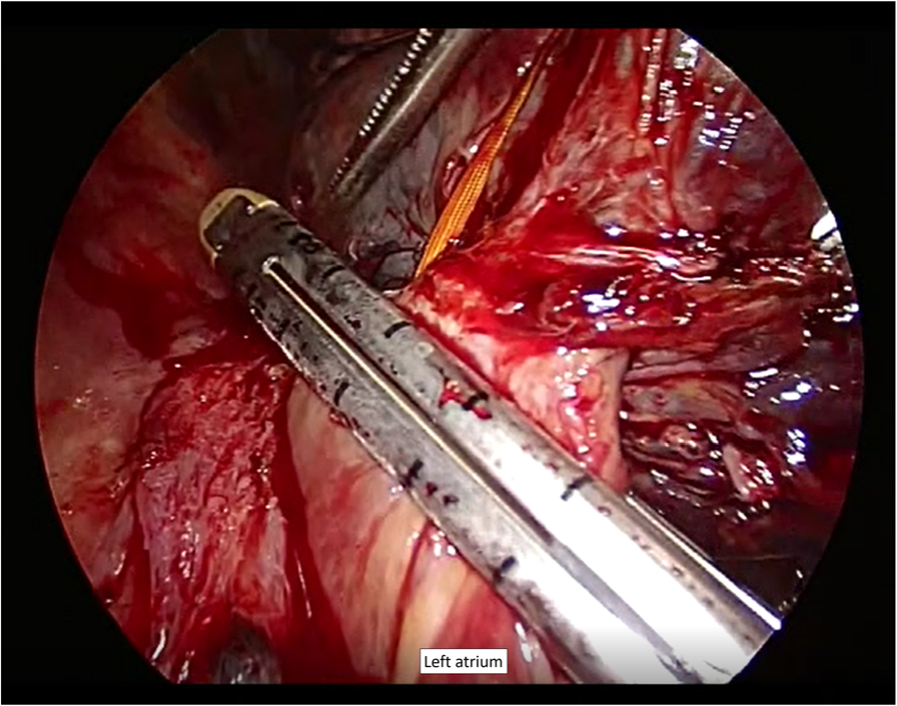
Resection of the left atrium with a tri-stapler

**Fig. 3:**
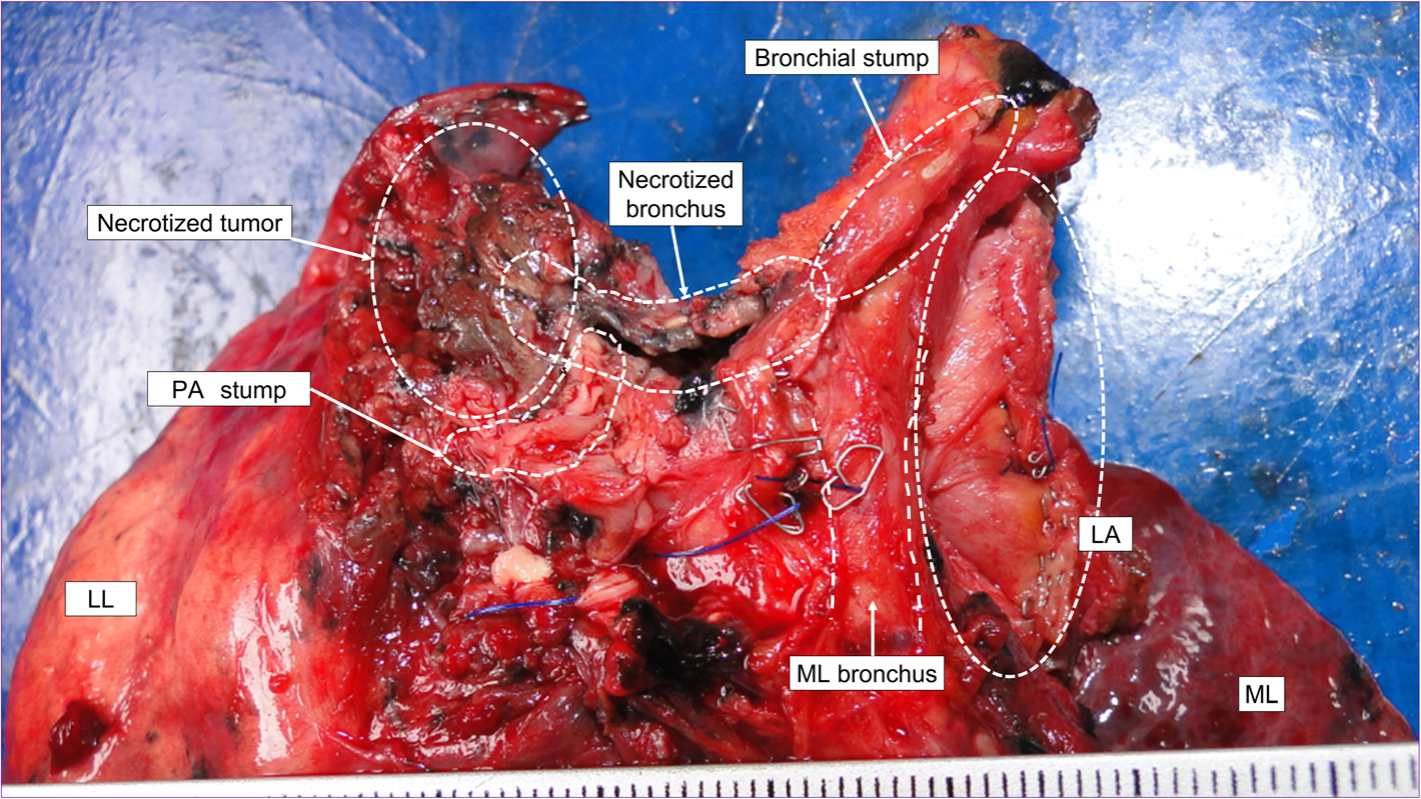
Resected specimen. The bronchial stump can be observed close to the necrotized bronchi. LN, lymph node; PA, pulmonary artery; ML, middle lobe; LL, left lower lobe; LA, left atrium

Right thoracotomy revealed severe scar formation in the hilar region, and a shrunken tumor was palpated at the hilum beneath the bifurcation of the upper lobe bronchus. No direct tumor invasion into the intrapericardial space or left atrial wall was found through pericardiotomy. A tourniquet was positioned around the right main pulmonary artery to facilitate safer bleeding control. The origin of the right inferior pulmonary vein was surrounded by dense adhesion and scar tissue. We extended the pericardiotomy to the dorsal side to widely expose the left atrium and perform partial resection with an adequate safety margin (Fig. 2). This was performed using an automatic stapler (Signia™ Stapling System; Medtronic, Dublin, Ireland) with a tri-staple curved tip camel 60 cartridge (Endo GIA™ 60; Medtronic). Upon clamping the left atrium with the cartridge, no error message was displayed on the automatic stapling system, and no change in firing speed was observed. As pulmonary artery could not be exposed, it was transected with surrounding scar tissue beneath the apicoposterior branch under clamping the right main pulmonary artery and closed with running polypropylene suture. The bronchial wall was necrotized from the distal portion of the intermediate bronchus to the B6 segmental bronchus. The proximal intermediate bronchi were isolated and divided using the automatic stapler. However, the bronchial stump was close to the necrotized bronchus (Fig. 3) and was entirely included in the radiation field. The omental flap, which was well vascularized by the right gastro-omental artery, was transported into the right hemithorax through the diaphragm. The bronchial stump and pericardial defect were covered with the omental flap.

Pathological analysis revealed complete response to CRT with no viable cancer cells in the necrotized tumor and lymph nodes. The resected specimen showed fistula formation between the intermediate bronchus and necrotic cavity in the tumor. The existence of cancerous keratin pearl-like structures suggested squamous cell carcinoma.

The postoperative course was uneventful. The patient is alive without recurrence. A CT scan taken at 10 months post-surgery showed that both the bronchial stump and pericardial defect were tightly covered by the omental flap with sufficient volume and blood supply.

## Discussion

We successfully performed surgery to avoid fatal complications and secure radicality after definitive CRT for c-stage IIIB NSCLC. Extending pericardiotomy to the dorsal side to widely expose the left atrium and resecting the left atrium using an automatic tri-stapling system with adaptive firing technology enabled safe, accurate, and prompt suturing and dividing. We used an omental flap to prevent bronchopleural fistula.

This patient did not complicate major cavitation associated with massive hemorrhage on the baseline CT, as described by Ito et al. [1]. However, we emphasize the risk of developing fatal hemorrhage or fistula formation due to tumor necrosis, although the response to the CRT treatment increased the potential for complete resection.

Some literatures reported the use of the Sondergaard technique for left atrial resection to avoid using cardiopulmonary bypass and enable increased margin of resection by performing interatrial groove dissection [5, 6]. Meanwhile, in cases where tumor progression is localized to the origin of the pulmonary vein, like that of our patient, partial atrial resection is performed with a simple atrium clamping technique [7]. In cases where the tumor invades down the origin of the pulmonary vein, and the left atrial wall is tumor free, partial left atrium resection is required to ensure an adequate margin. In these cases, using a stapler is an alternative method to perform left atrium resection. Galvaing et al. reported that partial resection of the left atrium was performed either on clamp or with an automatic staple although left atrium partial resection with tri-stapler has not been described in detail to date [6]. We believe using an automatic stapler for left atrial resection is associated with various advantages: a smaller margin is required for suturing, which enables the operator to secure an adequate safety margin; it avoids the risk of massive bleeding due to dislocation of the clamp forceps while suturing the atrium; it enables accurate and prompt suturing and dividing. However, the stapler should be used with caution, as it may lead to a catastrophic rupture of the left atrium when a stapler malfunctions. Tunezuka et al. reported that they had experienced massive bleeding along the staple line after dividing the left atrium with a stapler [8]. To avoid such risks, surgeons must be prepared to be able to apply forceps and place repair sutures with felt pledgets.

Galvaing et al. described using Endo GIA universal stapler with green cartridges. In our case, we chose the camel cartridge [6]. According to the product information, green cartridges should be used on tissue that can comfortably compress to 2 mm, whereas camel cartridges should be used on tissue that can comfortably compress to 0.88–1.88 mm. Based on the intraoperative findings, we thought that the camel cartridge would be suitable for this patient, who has a relatively small physical size (159 cm tall with body weight of 64 kg) with normal atrial muscle (no hypertrophy or atrophy). Of note, the Adaptive Firing Technology™ that measures the firing force and automatically adjusts the stapler speed of the Signia™ Stapling system (Medtronic) assists in determining the appropriate cartridge because it gives an alarm message if an inadequate cartridge is chosen.

Bronchopleural fistula is one of the most serious postoperative complications of post-CRT surgery. A higher rate of bronchopleural fistula is expected because the necrotized region is close to the bronchial stump along with a post-CRT hypovascular status. Vascularized flaps, such as the intercostal muscle flap, pericardial fat pad, and omental flap, are used to cover the bronchial stump [3]. Of these, we used an omental flap because it has a rich vascular supply and immunologic action and was located outside the radiation field.

## Conclusion

We performed lower bilobectomy in a patient with c-stage IIIB NSCLC to prevent fatal bleeding due to tumor cavitation during definitive CRT. An automatic tri-stapler facilitated the resection of the left atrium, and the patient has been alive without major complications or tumor recurrence for 10 months after the surgery. Optimal approaches for stage IIIB NSCLC or post-CRT surgery remain controversial. However, in a case with a chance of complete (R0) resection, when the risk of developing fatal complications might outweigh the risk of post-CRT surgery perioperative complications, surgery should be considered as a treatment option.

## Data Availability

Data sharing is not applicable to this article as no datasets were generated or analyzed during the current study.

## Abbreviations

CRT: Chemoradiotherapy
CT: Computed tomography
LA: Left atrium
LL: Left lower lobe
LN: Lymph node
ML: Middle lobe
NSCLC: Non-small cell lung cancer
PA: Pulmonary artery
